# Role of innate lymphoid cells and dendritic cells in intradermal immunization of the enterovirus antigen

**DOI:** 10.1038/s41541-019-0108-6

**Published:** 2019-03-27

**Authors:** Shengtao Fan, Yun Liao, Yaru Lian, Guorun Jiang, Li Jiang, Chenhong Dong, Erxia Yang, Lichun Wang, Xingli Xu, Min Feng, Ying Zhang, Qihan Li

**Affiliations:** 1 Institute of Medical Biology, Chinese Academy of Medical Sciences & Peking Union Medical College, Yunnan Key Laboratory of Vaccine Research and Development on Severe Infectious Diseases, 650118 Kunming, Yunnan China; 2 Aimei Convac BioPharm (Jiangsu) Co., Ltd., 225300 Taizhou, Jiangsu China

**Keywords:** Viral infection, Inactivated vaccines, Inactivated vaccines

## Abstract

Enterovirus type 71 (EV71) and coxsackievirus A 16 (CA16) are the major pathogens of human hand, foot, and mouth disease (HFMD). In our previous study, intramuscular immunization with the inactivated EV71 vaccine elicited effective immunity, while immunization with the inactivated CA16 vaccine did not. In this report, we focused on innate immune responses elicited by inactivated EV71 and CA16 antigens administered intradermally or intramuscularly. The distributions of the EV71 and CA16 antigens administered intradermally or intramuscularly were not obviously different, but the antigens were detected for a shorter period of time when administered intradermally. The expression levels of NF-κB pathway signaling molecules, which were identified as being capable of activating DCs, ILCs, and T cells, were higher in the intradermal group than in the intramuscular group. Antibodies for the EV71 and CA16 antigens colocalized with ILCs and DCs in skin and muscle tissues under fluorescence microscopy. Interestingly, ILC colocalization decreased over time, while DC colocalization increased over time. ELISpot analysis showed that coordination between DCs and ILCs contributed to successful adaptive immunity against vaccine antigens in the skin. EV71 and/or CA16 antigen immunization via the intradermal route was more capable of significantly increasing neutralizing antibody titers and activating specific T cell responses than immunization via the intramuscular route. Furthermore, neonatal mice born to mothers immunized with the EV71 and CA16 antigens were 100% protected against wild-type EV71 or CA16 viral challenge. Together, our results provide new insights into the development of vaccines for HFMD.

## Enterovirus vaccine: Intradermal immunization improves protection

Coxsackievirus A 16 (CA16) and enterovirus 71 (EV71) infections are the most common cause of hand-foot-and-mouth diseases. Inactivated virus has been evaluated as potential vaccine for both viruses in animal models, but protection was only achieved for EV71. In this study, led by Qihan Li from the Chinese Academy of Medical Sciences, researchers show that intradermal, as compared to intramuscular immunization, results in an elevated immune response and improved protection from EV71 and CA16 infection in mice. Intradermal vaccination increases interaction of vaccine antigen with dendritic cells and innate lymphoid cells at the site of inoculation, as compared to intramuscular vaccination. Intradermal vaccination furthermore improves the antibody and T cell response and protects mice from infection. However, complete protection of mice from CA16 infection was only achieved after intradermal immunization with a combination of inactivated EV71 and CA16 vaccine, suggesting that further improvements of this vaccine candidate will be necessary.

## Introduction

Previous studies of human enterovirus vaccines have substantially improved our knowledge of the immunology of viral attenuated and inactivated vaccines and advanced our understanding of vaccine mechanisms.^1^ The application of the attenuated oral poliomyelitis vaccine (OPV) worldwide suggests that viral attenuated vaccines are capable of eliciting specific immune responses with effective clinical protection against viral challenge via immunizing the gut epithelium, which is a natural infection route for poliovirus.^2,3^ However, the immunological effect achieved by the inactivated poliovirus vaccine in children requires large amounts of antigen and a proper immunization schedule, which has been improved continuously since its application in the 1990s.^4^ The global action plan for poliomyelitis eradication that was drafted by the World Health Organization (WHO) recommended using the inactivated poliovirus vaccine of the Sabin strain (sIPV) in developing countries; however, concerns were raised regarding the immune patterns of sIPV, and intradermal inoculation was suggested to achieve better immune effects with smaller amounts of antigen.^5,6^ In fact, some data on intradermal immunization with IPV were positive and indicated that immunological studies would be useful for the development of novel inactivated enterovirus vaccines.^7^ Recently, basic immunological studies have provided not only knowledge about the sensing of pathogenic antigens by pattern recognition receptors (PRRs) in epithelial tissues and the coordination of various innate immune cells for antigen presentation to T cells but also data on the systematic mechanism of adaptive immunity that is activated by comprehensive signals from the innate immune response.^8,9^ These studies described various groups of dendritic cells (DCs), which can perform antigen presentation upon activation, and reported that this process involves a type of innate immune cells termed innate lymphoid cells (ILCs).^10^ ILCs are classified into three groups that are located mainly in epithelial tissues and are rarely found in lymph nodes and other tissues; studies have suggested that ILCs can be activated by specific innate immune signals produced from infected epithelial tissues and subsequently secrete immune molecules to modulate DC activity and coordinate adaptive immune response activation.^11,12^ With each group of ILCs showing characteristic expression profiles for specific cytokines and cellular transcriptional factors that are involved in their activation and immunological activities,^13^ these cells are capable of providing qualitative indicators to distinguish innate immunity and the associated adaptive immune response after stimulation with a vaccine antigen.^14^ Based on these findings, we hypothesize that immune responses with varying characteristics that are elicited by different enterovirus antigens might be utilized to design specific vaccines. Enterovirus type 71 (EV71) and coxsackievirus A 16 (CA16), which are both major pathogens of human hand, foot, and mouth disease (HFMD), were deemed capable of inducing a systemic, clinical, and pathogenic response based on their ability to infect the epithelium of the respiratory or alimentary tract.^15,16^ However, studies of vaccine development with these two viruses suggested that the immunization provided by intramuscular inoculation of the inactivated EV71 vaccine in mice and macaques elicited effective immunity with clinical protection against viral challenge,^17^ while the immunization provided by inoculation of the inactivated CA16 vaccine via the same route in macaques was not effective, especially in viral challenge tests.^18^ This interesting immunological difference induced by two inactivated viral antigens that possess similar structural characteristics was addressed by analyzing the innate immune response, especially the responses of DCs and ILCs and the activation of adaptive immunity. The work described herein focused on the innate immune response elicited by the inactivated EV71 and/or CA16 antigen via the intradermal route compared to that elicited by these viral antigens via the intramuscular route. The results suggested that inoculation of the two viral antigens via the intradermal route, which might mimic the natural infectious pathway of the virus, is capable of activating an innate immune response, and innate immune activation of adaptive immunity, in which ILCs and DCs might play an important role through coordinated effects, was observed.

## Results

### Distributions of the EV71 and CA16 antigens in various inoculated tissues and the associated innate response

**Fig. 1:**
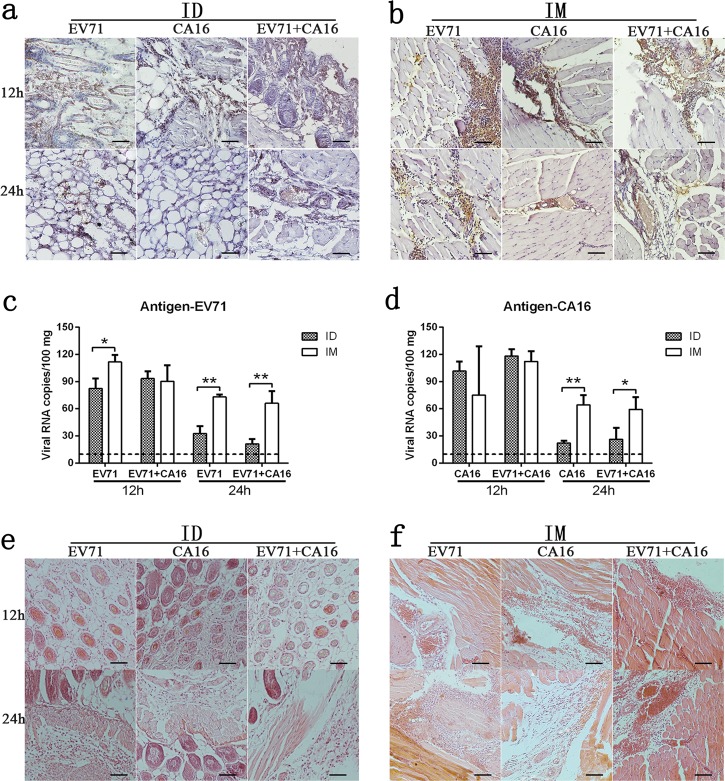
EV71 and CA16 antigen distribution in various tissues and pathological features. Immunohistochemical observation of the EV71 and CA16 antigens after administration via **a** intradermal (ID) and **b** intramuscular (IM) routes at 12 h and 24 h post inoculation. **c** EV71 viral RNA and **d** CA16 viral RNA in local tissues of three groups. Pathological observation of **e** intradermal (ID) tissues and **f** intramuscular (IM) tissues. Scale bar is 100 µm. Data are representatives of three independent experiments (error bars represent SD).; Statistical significance were assessed by unpaired *t* tests (*p < 0.01, **p < 0.001)

Previous data on vaccine immunization provided the general idea that inoculation into muscle tissue might not only maintain antigen aggregation in local tissues longer than observed for other routes to induce a constant immune stimulus but also decrease the risk of adverse vaccine effects.^19,20^ In the current study, immunological differences between the inactivated EV71 and CA16 antigens, which were administered with an aluminum adjuvant, were investigated. Mice were immunized with three groups of antigens, EV71, CA16, and EV71 combined with CA16, via the intramuscular or intradermal route. The antigen distributions in local tissues after inoculation via the intradermal or intramuscular route were not obviously different among the three groups (Fig. 1); however, the antigens delivered intradermally resided in the tissues for a shorter time than those delivered via the intramuscular route (Fig. 1a, b). At 24 h post inoculation, less antigen staining was observed in the intradermal tissues than in the intradermal tissues (Fig. 1a, b), and similar differences in the viral genomic RNA loads detected in skin and muscle tissue samples were observed (Fig. 1c, d). Furthermore, histopathological examination of the inoculated skin tissues from each group indicated slight inflammatory cell aggregation in intradermal tissue, similar to that observed in muscle tissue (Fig. 1e, f). These results imply a logical relationship between the antigen inoculation route and the innate response to the stimulus in the inoculated tissue, which was consistent with previous reports.^21^

### Expression profiles of innate immune signaling molecules in local tissues inoculated with the EV71 and/or CA16 antigens

**Fig. 2:**
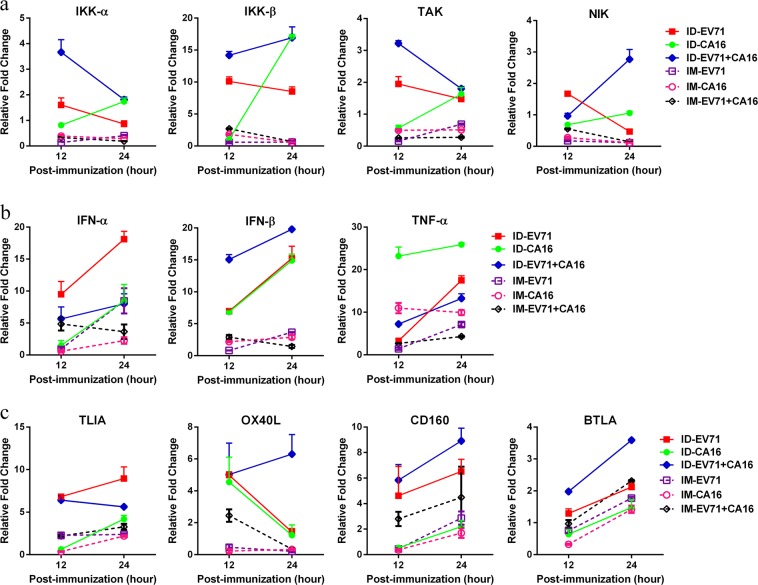
Expression profiles of signaling molecules in peripheral tissues. **a** IKKα, IKKβ, TAK, and NIK; **b** IFNα, IFNβ, and TNFα, and **c** TLIA, OX40L, CD160, and BTLA were detected in tissues from mice inoculated with EV71, CA16, or EV71 combined with CA16 via the intradermal (ID) or intramuscular (IM) route. All data for each control group (IM, ID) were treated as reference values to calculate the relative fold change. Data are representatives of three independent experiments (error bars represent SD)

The generation of an adaptive immune response after the vaccine antigen-induced activation of innate immunity in peripheral tissues usually depends on the dynamic transfer of various immune signaling molecules that originate from multiple sources, especially innate immune cells.^22,23^ Understanding this process might help identify potential indicators of various immunogenic signals that reflect a series of events in a dynamic immune response process and may be useful for analyzing the immunity induced by vaccines.^24,25^ Based on our current aim to investigate differences in the immunological characteristics of the EV71 and CA16 antigens after administration via different inoculation routes, we used q-RT-PCR to observe the expression profiles of various immunogenic molecules that function in activating ILCs in tissues in which both antigens were distributed. The IKKα, IKKβ, TAK, and NIK kinases are involved in NF-κB pathway signal transduction, which is a key transcriptional component of activated innate immunity; these kinases were expressed at higher levels in the intradermal group than in the intramuscular group (Fig. 2a). IFNα, IFNβ, and TNFα, which are immune molecules with antiviral capacity, showed the same tendency, exhibiting higher expression in the intradermal group than in the intramuscular group at 12 and 24 h post inoculation (Fig. 2b). Molecules capable of activating DCs, ILCs, and T cells, including TLIA, OX40L, CD160, and BTLA, showed varying levels of expression in the intradermal and intramuscular groups, but most of these factors were expressed at higher levels in the intradermal group (Fig. 2c). These results suggested that different EV71 and/or CA16 antigen immunization patterns are capable of inducing varying expression profiles of innate immune signaling molecules in local inoculated tissues.

### Relationship between the EV71 and/or CA16 antigens and ILCs in the tissues of mice immunized via the intradermal or intramuscular route

**Fig. 3:**
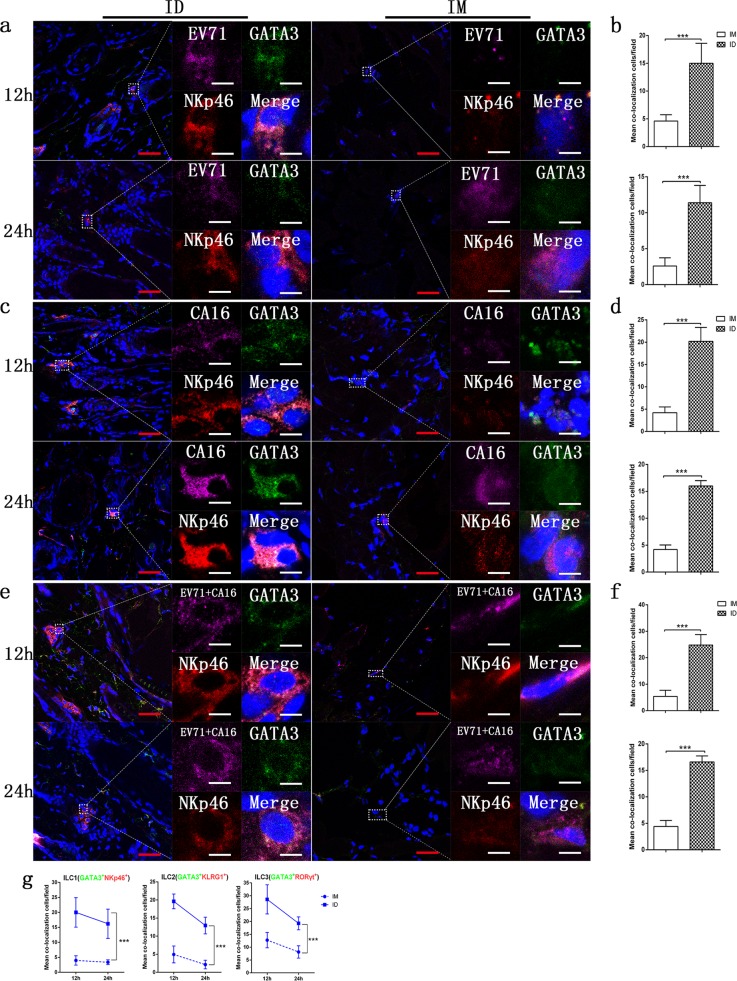
Relationships between the EV71 or CA16 antigen and ILC1 cells in local tissues post inoculation. **a** Representative confocal fluorescence images of EV71 expression (purple), GATA3 (green) and NKp46 (red) after intradermal (ID) or intramuscular (IM) administration of EV71 antigen at 12 and 24 h post inoculation. **c** Representative confocal fluorescence images of CA16 expression (purple), GATA3 (green) and NKp46 (red) after intradermal (ID) or intramuscular (IM) administration of the CA16 antigen at 12 and 24 h post inoculation. **e** Representative confocal fluorescence images of EV71+CA16 expression (purple), GATA3 (green) and NKp46 (red) at 12 and 24 h post inoculation of the EV71+CA16 antigens via the intradermal (ID) or intramuscular (IM) route. Statistical analysis of colocalization with **b** EV71, **d** CA16, and **f** EV71+CA16 cells. **g** Colocalization rates of ILC1, ILC2, and ILC3 cells after intradermal (ID) or intramuscular (IM) inoculation. Representative fluorescent cells in the white rectangle are shown at ×20 magnification after confocal microscopy. Red scale bar is 100 µm, white scale bar is 5 µm. Data are representatives of three independent experiments (error bars represent SD). Statistical significance were assessed by unpaired *t* tests (***p < 0.0001)

Recent studies of ILCs suggested that these important cells are capable of migrating and aggregating to the antigen-inoculated tissue site upon receiving signals from epithelial cell immune molecules.^12^ Functionally, these activated ILCs have the capacity to secrete secondary signaling molecules, including IL-22, IL-13, IL-4, Areg, and LTα3, to activate or coordinate DCs and other immune cells, especially resident T cells in epithelial tissue, as they possess antigen phagocytosis and T cell presentation abilities.^11,26^ This process serves as the antigenic signal for the transition from activated innate immunity to adaptive immunity. Thus, it is reasonable to infer that a relationship exists between antigens and ILCs in inoculated epithelial tissue. Therefore, we first observed the colocalization of antigens with various subgroups of ILCs using immunofluorescence microscopy. Antibodies specific for characterized molecules of the three ILC subgroups and antibodies specific for the EV71 or CA16 antigen showed the same tissue localization in both the intradermal and intramuscular groups under fluorescence microscopy (Fig. 3a, c, e; Supplementary Fig. 1a, c, e; Supplementary Fig. 2a, c, e). Interestingly, these colocalization characteristics were associated with the time course of the experiment, as higher rates of colocalization were observed at 12 h than at 24 post inoculation, showing a gradually decreasing tendency. However, observations of 50 fields randomly counted under a fluorescence microscope showed not only that the rates of antigen and ILC colocalization in the tissues gradually decreased over time post inoculation (Fig. 3b, d, f; Supplementary Fig. 1b, d, f; Supplementary Fig. 2b, d, f) but also that the rates in the intradermal groups were obviously higher than those in the intramuscular groups (Fig. 3g). These results suggested a role for antigen recognition in ILC phagocytosis, which presented as a quick and transient reaction and occurred more often in epithelial tissue than in muscle tissue.

### Relationship between the EV71 and/or CA16 antigen and DCs in the tissues of mice immunized via the intradermal or intramuscular route

**Fig. 4:**
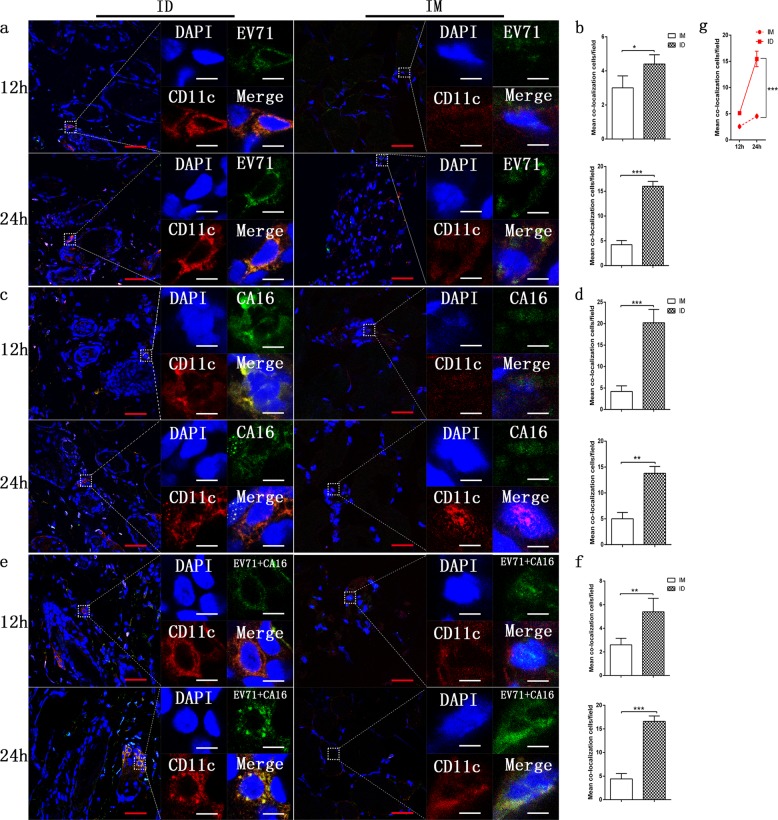
Relationship between the EV71 and/or CA16 antigen and DCs in tissues. **a** Representative confocal fluorescence images of EV71 expression (green) and CD11c (red) at 12 h and 24 h post inoculation of the EV71 antigen via the intradermal (ID) or intramuscular (IM) route. **c** Representative confocal fluorescence images of CA16 expression (green) and CD11c (red) at 12 and 24 h post inoculation of the CA16 antigen via the intradermal (ID) or intramuscular (IM) route. **e** Representative confocal fluorescence images of EV71+CA16 expression (green) and CD11c (red) at 12 and 24 h post inoculation of the EV71+CA16 antigen via the intradermal (ID) or intramuscular (IM) route. Statistical analysis of colocalization with **b** EV71, **d** CA16, **f** EV71+CA16 cells. **g** Colocalization rates of DCs after intradermal (ID) or intramuscular (IM) inoculation. Representative fluorescent cells in the white rectangle are shown at ×20 magnification after confocal microscopy. Red scale bar is 100 µm, white scale bar is 5 µm. Data are representatives of three independent experiments (error bars represent SD). Statistical significance were assessed by unpaired *t* tests (*p < 0.01, **p < 0.001, ***p < 0.0001)

Previous immunological studies successfully identified DCs as important immune cells that exert mediating effects between innate and adaptive immune responses and are capable of taking up antigens upon activation by specific signaling molecules and presenting them to T cells associated with regulatory cytokines and chemokines.^27,28^ Recent data further suggested that DCs are actually harmonized by various immunogenic signaling molecules from stimulated epithelial cells and activated ILCs, and they thus play a role together with ILCs to systematically activate adaptive immunity.^29,30^ Based on these data and considering the interrelation of both antigens with ILCs, we further observed the relationship between the EV71 and CA16 antigens and DCs in mice immunized via the intradermal or intramuscular route. Antibodies specific for the EV71 and CA16 antigens colocalized with the CD11c antibody on the DC surface in the skin and muscular tissues of mice immunized by the two routes, as determined by fluorescence microscopy (Fig. 4a–f). However, the rates of colocalization tended to be positively related to the experimental time course, with an increase over time (Fig. 4a–f), a trend that was opposite to that observed for the antigens and ILCs. Counts of the colocalized cells in 50 fields revealed this characteristic gradient increase over the time course of the experiment (Fig. 4g) and showed obviously higher rates of colocalized cells in skin tissue than in muscular tissue (Fig. 4g). These results suggest a possible logical affiliation of activated ILCs and functional DCs over time.

### ILC and DC coordination activates T cell proliferation more effectively

**Fig. 5:**
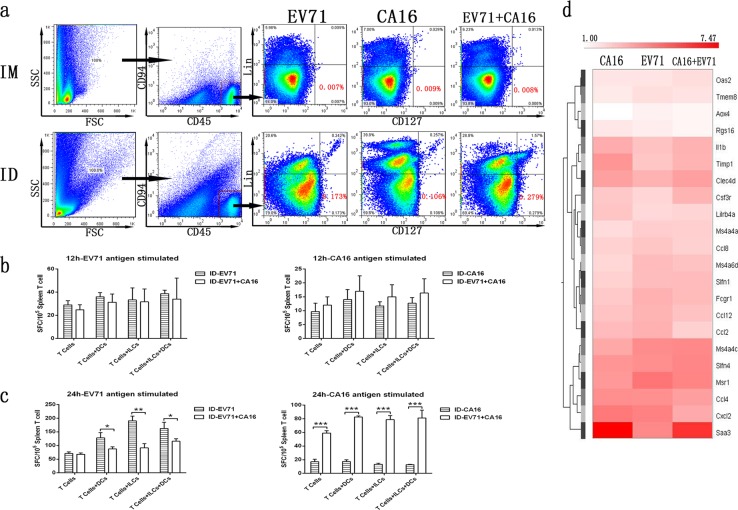
ILC and DC coordination is capable of enhancing T cell proliferation. **a** Flow cytometric analysis and comparison of CD94^−^CD45^+^CD127^+^Lin^−^ (ILCs) from skin and muscle at 12 h post inoculation. Data are representative of 3 separate experiments for each subset. **b** Specific IFN-γ proliferation of cocultured T cells and ILCs, T cells and DCs, and T cells, ILCs and DCs stimulated with EV71 antigen (left panel) and CA16 antigen (right panel) at 12 h post inoculation. **c** Specific IFN-γproliferation of cocultured T cells and ILCs, T cells and DCs, and T cells, ILCs and DCs stimulated with EV71 antigen (left panel) and CA16 antigen (right panel) at 24 h post inoculation. **d** mRNA expression profiles of skin from each group at 12 h post inoculation. All data from each control group (IM, ID) were treated as reference values to calculate the relative fold change. Data are representatives of three independent experiments (error bars represent SD). Statistical significance were assessed by unpaired *t* tests (*p < 0.01, **p < 0.001, ***p < 0.0001)

Classic immunological data suggest that the proliferation of specific reactive T cells during an adaptive immune response depends on antigenic presentation by innate immune cells, including DCs, which have a strong presenting capacity, and macrophages and B cells, which have a weaker presenting capacity.^31^ However, recent systematic immunological studies further suggested that activated ILCs are capable of presenting antigens to T cells, as they can coordinate with the function of DCs by secreting specific cytokines and other signaling molecules.^32,33^ This suggestion led to the hypothesis that DC and ILC coordination helps to successfully establish an adaptive immune response against a vaccine antigen. Based on this viewpoint, we attempted to isolate ILCs and DCs from skin or muscle tissues inoculated with the EV71 and/or CA16 antigen to further understand their roles in immune responses. Unfortunately, both ILCs and DCs were difficult to harvest from muscle tissue and were more easily harvested from skin tissue (Fig. 5a). The ILCs and DCs isolated from the skin at 12 and 24 h post inoculation were cultured with T cells isolated from the spleen of the same mouse or cultured alone for 12 h. These treated T cells were stimulated by the antigens, and their specific proliferation abilities were detected using an ELISpot assay based on IFN-γ specificity. T cells isolated from the spleens of mice immunized with the EV71 and/or CA16 antigen cultured alone and T cells cocultured with ILCs, DCs or ILCs+DCs isolated from the skin of the same mice at 12 h post inoculation showed similar proliferation levels after stimulation with antigen (Fig. 5b). Interestingly, for T cells isolated from mice immunized with the EV71 antigen at 24 h, the ELISpot results for both T cells alone and T cells cocultured with ILCs, DCs, or ILCs+DCs showed an obviously increased proliferative ability after specific antigen stimulation compared to that of other T cells (Fig. 5c). However, the T cells isolated from mice immunized with the CA16 antigen alone at 24 h showed a weaker proliferative response regardless of their coculture with DCs or ILCs, and the cells isolated from mice immunized with the EV71+CA16 antigens showed a stronger proliferative response after CA16 or EV71 antigen stimulation in the ELISpot assay (Fig. 5c). To investigate the mechanism underlying these differences, we analyzed the mRNA expression profiles of dermal tissues harvested from mice immunized intradermally at 24 h post inoculation. Some genes involved in the innate immune response, such as the chemokine ligands CCL2, 4, 8, and 12, Cxcl2, and IL-1β, showed varying levels of upregulation in the three groups compared to those in the mock-treated group (Fig. 5d). Among these genes that were expressed at various levels, some showed lower expression in the CA16 alone group than in the EV71 alone or EV71+CA16 group (Fig. 5d), but this trend was reversed for other genes (Fig. 5d). Whether this differential expression underlies the weaker T cell proliferation response after CA16 stimulation compared to that induced after EV71 stimulation requires further analysis; however, these observations do support our hypothesis.

### Adaptive immunity is induced in mice immunized with the EV71 and/or CA16 antigen via the intradermal or intramuscular route

**Fig. 6:**
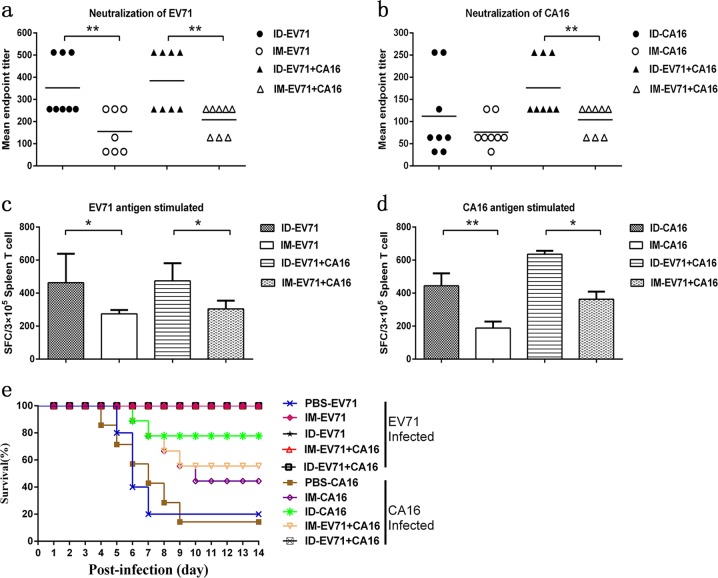
Immunological evaluation of mice immunized with the EV71 and/or CA16 antigen. Serum samples were tested for neutralization of **a** EV71 and **b** CA16 in each group. Each symbol represents one mouse, and the line indicates the mean titer value of each group. Specific IFN-γ proliferation abilities of spleen cells stimulated with the **c** EV71 or **d** CA16 antigen. Each group of 1-day-old BALB/c mice was challenged with EV71/FY23 or CA16/G20 to test the protective efficacy of adaptive immunity. The neonatal mice were monitored daily for survival for two weeks. Data are representatives of three independent experiments (error bars represent SD). Statistical significance were assessed by unpaired *t* tests (*p < 0.01, **p < 0.001)

Our previous study indicated that the inactivated EV71 vaccine was capable of inducing specific serum neutralizing antibody responses and cellular immune responses in rhesus models and humans,^17^ and these responses were associated with clear clinical protective efficacy.^34^ However, a study of inactivated CA16 vaccines in macaques did not report similar results, as no positive clinical protection was observed in viral challenge tests.^15^ In the current work, the intradermal route, which was inferred to be capable of inducing a coordinated response by DCs and ILCs to activate T cell proliferation, and the intramuscular route were used to investigate the differences in immune responses elicited by the two viruses. Based on the identified activation of innate immunity and its interrelation with T cell proliferation in mice immunized with the EV71 and/or CA16 antigen, classic immunological indicators of neutralizing antibodies and antigen-specific T cell responses with IFN-γ specificity were detected. In addition, a test of protection in neonatal mice was carried out by viral challenge to evaluate adaptive immunity against the EV71 and CA16 antigens. EV71 antigen immunization via the intradermal route was capable of significantly increasing the neutralizing antibody titer compared to that in mice immunized via the intramuscular route (Fig. 6a, b). CA16 antigen inoculation alone by the intradermal route also induced higher titers of neutralizing antibody than those achieved by the intramuscular route (Fig. 6c, d), and a significant increase in the neutralizing antibody titer against CA16 was observed in mice immunized with EV71+CA16 via the intradermal route compared to that in mice immunized via the intramuscular route (Fig. 6c, d). All groups, including those inoculated with EV71, CA16, or EV71+CA16 and those inoculated via the intradermal or intramuscular route, exhibited a significant specific-T-cell response in the ELISpot assay based on INF-γ specificity (Fig. 6e, f). Furthermore, a test of protection against viral challenge in neonatal mice born to immunized mothers revealed a 100% protection rate against the EV71 and CA16 viruses in the EV71+CA16 immunization group administered via the intradermal route and the EV71 alone groups administered via the intradermal and intramuscular routes (Fig. 6g). Immunization with the CA16 antigen alone did not show the same protective efficacy (Fig. 6g).

## Discussion

Two conclusions from previous studies performed using oral attenuated and inactivated poliovirus vaccines are helpful for our current work: mimicking the natural infectious route might optimize the immunity elicited by a vaccine,^35^ and the formulation and administration of the inactivated vaccine should be optimized individually based on the characteristics of the immunity induced by the vaccine.^36^ Thus, our studies of the inactivated EV71 and CA16 vaccines were designed based on previous data obtained from studies of viral pathogenesis in rhesus macaques.^15,37^ The results obtained in these studies suggested that the inactivated EV71 vaccine is capable of inducing immunity with clinical protective efficacy, while the CA16 vaccine is not^17,18^; these findings improved our understanding of various immunogenic pathways. These data indicated that EV71 and CA16, two members of the enterovirus family, are capable of infecting humans by entering epithelial cells of the respiratory and/or alimentary tract and then replicating their viral genomes,^16^ and this event serves as a source of pathogen-associated pattern molecules (PAMPs) that can activate cellular PRRs.^38,39^ The outcome of this event is an epithelial innate immune response, which presents as transcriptional induction of the NF-κB system and the expression of various immune signaling molecules.^23^ These signaling molecules are capable of transferring antigenic stimuli to DCs, ILCs, and other innate immune cells and coordinating their migration and aggregation to infectious sites, leading to a local inflammatory reaction.^10^ While this process is typically observed in epithelial, muscular, mucosal, and other tissues, it occurs more often in epithelial tissue enriched with ILCs and DCs than in other tissues.^40^ Based on these data, it would be reasonable to infer that administration of the inactivated CA16 antigen via the intradermal route alone or together with the EV71 antigen might sequentially activate each innate and adaptive immunity component, potentially exerting a clinically protective effect similar to that induced by natural viral infection.^7,41^ Therefore, we aimed to identify a candidate immunogenic mechanism for the inactivated CA16 vaccine by mechanistically analyzing viral antigen-mediated activation of the innate immune system and the subsequent initiation of an adaptive immune response. Herein, we observed how the EV71 and CA16 antigens interacted with ILCs and DCs and compared the abilities of these immunization strategies to transfer an antigenic stimulus to T cells in epithelial and muscle tissues. The results suggested that inoculation of EV71 and/or CA16 into skin tissue was capable of eliciting the expression of various immune signaling molecules, most of which were determined to be related to the activation of ILCs and DCs,^42^ and interactions between viral antigens and subgroups of ILCs were observed more often in epithelial tissue than in muscle tissue. Although these reactive features were found in muscle tissue inoculated with the same antigens, the significant quantitative difference in their occurrence in both tissue types might underlie the disparate outcomes. Interestingly, the interaction between the viral antigens and ILCs, as indicated by colocalization under a fluorescence microscope, was increased at 12 h post inoculation and decreased over time, while the interaction between the viral antigens and DCs exhibited the opposite trend, increasing from 12 to 48 h post inoculation. This finding suggests that a logical relationship exists between the activation of ILCs and DCs. In an in vitro experiment involving T cells, ILCs, and DCs from homologously immunized mice, ILCs and DCs showed a coordinated ability to stimulate specific T cell proliferation, as T cells cultured with ILCs and DCs together showed a higher proliferative rate in response to the CA16 antigen in an ELISpot assay than T cells cultured alone in the EV71 + CA16-immunized group. Up- or downregulation of genes involved in innate immune activation was observed in the mRNA profiles of epithelial tissues obtained from inoculated mice, which showed differences in CA16-, EV71-, and EV71+CA16-immunized mice inoculated via the intradermal and intramuscular routes. Furthermore, the clinically protective effect against viral challenge in offspring born to immunized mice, especially those born to mice immunized with the EV71+CA16 antigens via the intradermal route, confirmed our above hypothesis. Our data regarding each critical reactive component of the immunogenic process stimulated by viral antigens suggest not only that a candidate combined EV71 and CA16 inactivated vaccine is capable of eliciting effective immunity upon inoculation via the intradermal route but also that the coordination of ILCs and DCs might play an important role in the immune response elicited by enterovirus antigens.

However, because our knowledge of ILCs is still scarce, this study clarified neither the details of the relationship between the EV71 and/or CA16 antigen and ILCs nor the mechanism underlying ILC and DC coordination during antigen presentation to T cells, although previously reported data indicated that ILCs are capable of presenting antigens to T cells.^32^ Therefore, identifying the mechanism that underlies ILC and DC coordination from multiple perspectives and using this information to develop a strategy for viral vaccine development requires further study. Nevertheless, our work furthers our understanding of the process by which vaccine antigens stimulate an innate immune response by eliciting the expression of immune signaling molecules and interacting with ILCs and DCs and elucidates the potential of the combined EV71 and CA16 inactivated vaccine.

## Methods

### Ethics statement

Female four-week-old BALB/c mice were purchased from Beijing Vital River Laboratory Animal Technology Co., Ltd. All mice were housed in a specific pathogen-free facility at the Institute of Medical Biology. The Yunnan Provincial Experimental Animal Management Association (approval No. SCXK [Dian]K2015-0006) and the institutional Experimental Animal Ethics Committee (approval No. YISHENGLUNZI[2016]4) approved the experimental protocols.

### Mouse study design

Female mice were randomly divided into the control, EV71, CA16, and EV71+CA16 groups. Inactivated vaccines were administered to 20 mice intramuscularly (IM) and 20 mice intradermally (ID) in each group. Each mouse was inoculated with the vaccine (Al(OH)_3_ adjuvant plus EV71: 100 U; CA16: 100 U; or EV71+CA16: 100 U+ 100 U) via one of the immunization routes. Three mice were sacrificed at 12 and 24 h after immunization, and skin and muscle tissues harvested from the injection site were used to assay viral load, cytokine levels, pathology, and immunofluorescence. Three mice were sacrificed on days 3 and 7 after immunization, and their spleens were collected for splenocyte isolation. Serum samples were collected at 28 and 56 days after immunization. After two immunizations, two female mice from each group were paired with naive male mice. Selected nests (*n* > 5) of newborn suckling mice were challenged with a wild-type strain via intracerebral injection within 24 h. The EV71 (nest = 3) and EV71 + CA16 (nest = 3) groups were infected with the wild-type strain FY23, while the CA16 (nest = 3) and EV71+CA16 (nest = 3) groups were infected with the wild-type strain G20. The newborn mice exhibited abnormal signs or symptoms for 15 days, and some of the mice died.

### Splenocyte transcriptome analysis

Splenocytes were isolated from cell suspensions by density gradient centrifugation using Ficoll.

Total RNA was extracted from the splenocytes using TRIzol according to the manufacturer’s instructions (Cat # 15596026, Invitrogen). The RNA quantity and integrity were evaluated using the Nano Drop system and a Bioanalyzer, and four miRNA libraries (CA16 group, EV71 group, CA16+EV71 group, and control) were constructed according to Illumina’s instructions and sequenced (Illumina MiSeq, Illumina, San Diego, CA, USA). Before further analysis, the raw data were evaluated using FastQC software (version 0.11.2), and the NOISeq method^43^ was used to analyze differentially expressed genes (DEGs). A false discovery rate (FDR) ≤ 0.001 and an absolute Log_2_ ratio value ≥ 1 were used to determine the significance of gene expression differences.

### Neutralization assay

The mouse serum samples were serially diluted two times and incubated with 100 TCID_50_ of the FY23 strain (EV71) or G20 strain (CA16) for 1 h at 37 °C. The end-point neutralization titers were determined by 50% plaque reduction assays using Vero cells at 37 °C in a 5% CO_2_ incubator.^44^

### Immunofluorescence and confocal microscopy

Skin tissues from immunized mice were collected and immediately frozen in liquid nitrogen. The tissue sections were embedded in OCT (Tissue-Tek OCT Compound 4583, Sakura) and sliced on a cryostat at a 5 µm thickness (CM1850, Leica) according to the manufacturer’s protocol. The sections were fixed in acetone for 10 min at −20 °C and then blocked using 5% bovine serum albumin (BSA). For detection of the EV71 or CA16 antigen, the sections were sequentially incubated with a primary rabbit anti-enterovirus 71 VP1 antibody (Cat # GTX132339, GeneTex Inc.) or a rabbit anti-COX A16 polymerase 3CD polyclonal antibody (Cat # bs-10066R, BIOSS) and an AlexaFluor 568-conjugated donkey anti-rabbit IgG secondary antibody (Cat # A10042, Invitrogen). DCs were detected with an anti-CD11c antibody (Cat # ab33483, Abcam) and the Alexa Fluor® 647-AffiniPure donkey anti-rat IgG secondary antibody (Cat # 712-001-003, Jackson). ILCs were detected with a goat anti-GATA-3 antibody (Cat # sc-269, Santa Cruz). The ILC1 population was detected with a mouse anti-NKp46/NCR1 antibody (Cat # MAB1850-100, R&D), the ILC2 population was detected with a mouse anti-human KLRG1 antibody (MAFA) (Cat # 367702, Biolegened), and the ILC3 population was detected with a mouse anti-RORγt antibody (Cat # 562663, BD); Alexa Fluor 488-conjugated AffiniPure donkey anti-goat IgG (Cat # 711-545-152, Jackson) and Alexa Fluor^®^ 647-AffiniPure donkey anti-rat IgG were used as secondary antibodies for ILC detection. All cell nuclei were detected with DAPI. Fluorescence was visualized and analyzed using a confocal microscope (TCS SP2, Leica).

### Cytokine analysis

Total RNA was extracted from the skin and muscle tissues of mice at various time points after immunization using TRIzol-A^+^ Reagent (Cat # DP421, Tiangen) according to the manufacturer’s protocol. RNA was amplified using the One Step TB Green™ Prime Script™ PLUS RT-PCR Kit (Cat # RR096A, TaKaRa). The primers used for the quantification of IKKα, IKKβ, TAK, NIK, IFNα, IFNβ, TNFα, TLIA, OX40L, CD160, and BTLA are shown in Supplementary Table 1.

### Histopathological and immunohistopathological analysis

Skin and muscle tissues from euthanized mice were fixed in 10% formaldehyde, dehydrated, embedded, and then cut into 4-μm-thick sections for hematoxylin and eosin (HE) staining assays. Pathological changes were examined by light microscopy (ECLIPSE Ti-s, Nikon). For immunohistochemical analysis, the sections were prepared according to the manufacturer’s protocol. Briefly, the sections were deparaffinized, hydrated, antigen-repaired, and then blocked in 4% BSA. The sections were then incubated with an anti-enterovirus 71 antibody and cross-reacted with coxsackievirus A 16 (Cat # MAB979, Millipore) overnight at 4 °C. The sections were then washed three times with PBS and stained with a goat poly-HRP anti-rabbit IgG antibody (Cat # AS040, ABclonal) for 35 min at 37 °C. Finally, the slides were examined under a light microscope.

### Detection of the EV71 and CA16 antigens at the immunological site

Skin and muscle tissues collected from immunized mice were homogenized using a Tissue Lyser II system (Qiagen, Hilden, Germany). Viral RNA was extracted from tissues using the Simply P Total RNA Extraction kit according to the manufacturer’s protocol. q-RT-PCR quantification was performed using the One Step PrimeScript RT-PCR Kit(Cat # RR066A,TaKaRa) on a BIO-RAD iCycler Thermal Cycler (Bio-Rad Laboratories, Inc., CA, USA). The primers and probes for EV71^37^ and CA16^18^ are shown in Supplementary Table 2.

### Experimental inactivation of the EV71 and CA16 vaccines

The inactivated EV71 vaccine (FY23)^45^ and the inactivated CA16 vaccine (KM/M08)^46^ were prepared in human diploid cells (KMB-17 strain). Briefly, the viruses were inactivated with 1:4000 formaldehyde at 37 °C for 72 h and concentrated 50-fold for Sepharose 6 Fast Flow (Amersham, USA) chromatography purification. The purified viruses emulsified into a 1 mg/ml Al(OH)_3_ adjuvant. The antigen content (100 U/ dose) was assessed by ELISA.

### Isolation of T cells from mouse spleens

Spleens were removed from euthanized mice and cut into 3-mm-thick sections. The tissue pieces were filtered through a 70 μm cell strainer using a plunger from a 5 ml syringe to obtain a single-cell suspension. The cell strainer was washed with 5 ml of PBS, and splenocytes were isolated from the cell suspension by density gradient centrifugation using Ficoll. The splenocytes were then resuspended in Hanks’ Balanced Salt Solution (HBSS) containing 2% fetal bovine serum (FBS), and the EasySep™ Mouse T Cell Isolation Kit (Cat # 19851, STEMCELL Technologies Inc) was used to isolate naive and highly purified T cells from mouse splenocytes by immunomagnetic negative selection according to the manufacturer’s instructions. Briefly, 1 × 10^8^ nucleated splenocytes/mL were placed into a round-bottom tube, and rat serum and an isolation cocktail were added. The mixture was incubated for 10 min at RT, and Rapid Spheres™ were then added to the sample. The tube was placed into the magnet and incubated for 2.5 min at RT. Unwanted cells were labeled with biotinylated antibodies and streptavidin-coated magnetic particles. The magnet was removed, and the enriched cell suspension was poured into a new tube in one continuous motion. The isolated cells were immediately available for coculture with ILCs or DCs.

### Isolation of lymphocytes from mouse skin and muscle

Back skin tissues containing the antigen injection sites were dissected from euthanized mice, placed into dishes and cut into 0.2 mm^2^ pieces with scissors. The pieces of tissue were transferred into 50 ml tubes and incubated with a digestion solution containing 5 mg/mL collagenase I (Cat # C0130, Sigma), 2.5 mg/mL trypsin (Cat # 27250018, ThermoFisher SCIENTIFIC) and 1 U/mL DNase I(Cat # AMPD1, Sigma) in Roswell Park Memorial Institute (RPMI) 1640 medium for 1 h at 37 °C with shaking at 200 rpm. The digested supernatant was filtered through a 70 μm cell strainer to obtain a single-cell suspension. Skin lymphocytes were isolated by centrifugation in Lymphoprep (Cat # LTS1092P, Haoyang), and thigh muscle cells were isolated from euthanized mice according to the methods described above.

### Flow cytometry analysis and cell sorting

Skin and muscle lymphocytes were washed three times with PBS, and 20 μl of a fluorophore-conjugated antibody (APC-CD94 (# 105512), PE/Cy5-CD127 (Cat # 135016), FITC-Lineage cocktail (Cat # 133301), and PE-CD45 (# 103106) purchased from Biolegened, PE/Cy7-CD11c (Cat # 561022) purchased from BD Pharmingen) was then added to cells resuspended at a concentration of 10^6^ cells/ml. The cells were stained for 30 min at 4 °C and washed twice prior to flow cytometric analysis (LSR Fortessa, BD) and sorting (Influx, BD). ILCs were identified from lymphocytes using the marker combination CD94^-^CD45^+^CD127^+^Lin^−^, and DCs were identified from lymphocytes using CD11c^+^.

### IFN-γ-specific ELISpot assay of T cell, ILC, and DC cocultures

An enzyme-linked immunospot (ELISpot) assay was performed to analyze interferon (IFN)-γ according to the manufacturer’s instructions (Cat # 3421M-4HPW-2, Mabtech). Briefly, after the plates were washed four times with PBS, the EV71, or CA16 antigen (20 U/well) and a cell suspension (5 × 10^5^ cells/well) were sequentially added, and the mixture was incubated for 36 h at 37 °C and 5% CO_2_. The cells were removed, and the plate was sequentially incubated with secondary antibodies and a substrate solution. The spots were inspected and counted using an automated ELISpot reader (CTL, OH, USA).

### Statistical analysis

All data are expressed as the mean ± SD. Significant differences between groups were analyzed by *t* tests (GraphPad Prism; GraphPad Software, San Diego, CA, USA), and *p* < 0.05 was considered statistically significant.

### Reporting Summary

Further information on experimental design is available in the Nature Research Reporting Summary linked to this article.

## Supplementary information

Reporting Summary

SUPPLEMENTAL MATERIAL

## Data Availability

The data that support the findings of this study are available from the corresponding author upon reasonable request.

## Supplementary information

**Supplementary Information** accompanies the paper on the *npj Vaccines* website (10.1038/s41541-019-0108-6).
