# Author Correction: Role of innate lymphoid cells and dendritic cells in intradermal immunization of the enterovirus antigen

**DOI:** 10.1038/s41541-019-0141-5

**Published:** 2019-11-04

**Authors:** Shengtao Fan, Yun Liao, Yaru Lian, Guorun Jiang, Li Jiang, Chenhong Dong, Erxia Yang, Lichun Wang, Xingli Xu, Min Feng, Ying Zhang, Qihan Li

**Affiliations:** 1 Institute of Medical Biology, Chinese Academy of Medical Sciences & Peking Union Medical College, Yunnan Key Laboratory of Vaccine Research and Development on Severe Infectious Diseases, 650118 Kunming, Yunnan China; 2 Aimei Convac BioPharm (Jiangsu) Co., Ltd., 225300 Taizhou, Jiangsu China

**Keywords:** Viral infection, Inactivated vaccines, Inactivated vaccines

**Correction to:** *npj Vaccines* 10.1038/s41541-019-0108-6, published online 27 March 2019

In the original version of this Article, the upper middle panel in Fig. 1f was mistakenly duplicated to the lower middle panel. This has now been corrected in the PDF and HTML versions of the Article.

