# Supplementary material for: Role of innate lymphoid cells and dendritic cells in intradermal immunization of the enterovirus antigen
Source: NPJ Vaccines. 2019 Mar 27;4:14. doi: 10.1038/s41541-019-0108-6 (PMC6437170; doi:10.1038/s41541-019-0108-6)
Supplement: Supplementary file 2 — SUPPLEMENTAL MATERIAL [file 41541_2019_108_MOESM2_ESM.pdf]

Supplementary Table 1. Primers used for quantitative RT-PCR in the study.

Primer name	Sequence (5'–3')
M-BTLA-F	GCCAGGACAGGAGAGTTA
M-BTLA-R	CTTACACCAAGTCACATTAGG
M-CD160-F	CCTGAGACCAACTTAGAACA
M-CD160-R	AACACCAACTGAGATGACTT
M-IKKα-F	TGGAAGAGACTGCTGACA
M-IKKα-R	ATGAAGAACACTTGCTGAGA
M-IKKβ-F	CTCCGAAGATACTTGAACCA
M-IKKβ-R	CGATGCGATGTCACTCAG
M-NIK-F	AGCAACTGGAGATAGAACTG
M-NIK-R	CTGAGGCAGGAGAGGATT
M-OX40L-F	TGCTTCTGTGCTTCATCTAT
M-OX40L-R	ATCTGGTAACTGCTCCTCT
M-TAK-F	CCATCACTTACACAGCAATC
M-TAK-R	CCTCACAGATACATACACAGA
M-TLIA-F	AATAAGCAACAACTGGTTCC
M-TLIA-R	ATTAGTCTGTCTCCTTCTTCC
M-TNFα-F	GTGGAACTGGCAGAAGAG
M-TNFα-R	GAGAAGAGGCTGAGACATAG
M-IFNα-F	CTTCCTCAGACTCATAACCT
M-IFNα-R	AGTCCTTCCTGTCCTTCA
M-IFNβ-F	AACTCCACCAGCAGACAG
M-IFNβ-R	GAGAGCAGTTGAGGACATC

Supplementary Table 2. Primers and probes used for detection of the EV71 and CA16 antigens in the study.

Name	Sequence (5'–3')
EV71 -F	CCCTGAATGCGGCTAATCC
EV71 -R	ATTGTCACCATAAGCAGCCA
EV71 –Probe	6FAM -CCAGCGGGTAGTGTGTCGTAACGGG- TAMRA
CA16-F	CAACCCATCTGTGTTTGTGAAAA
CA16-R	GGTATGCACTAGCTGGTGACATG
CA16-Probe	6FAM -CCGCCAGCTCAAGTGCAGTCCC-TAMRA

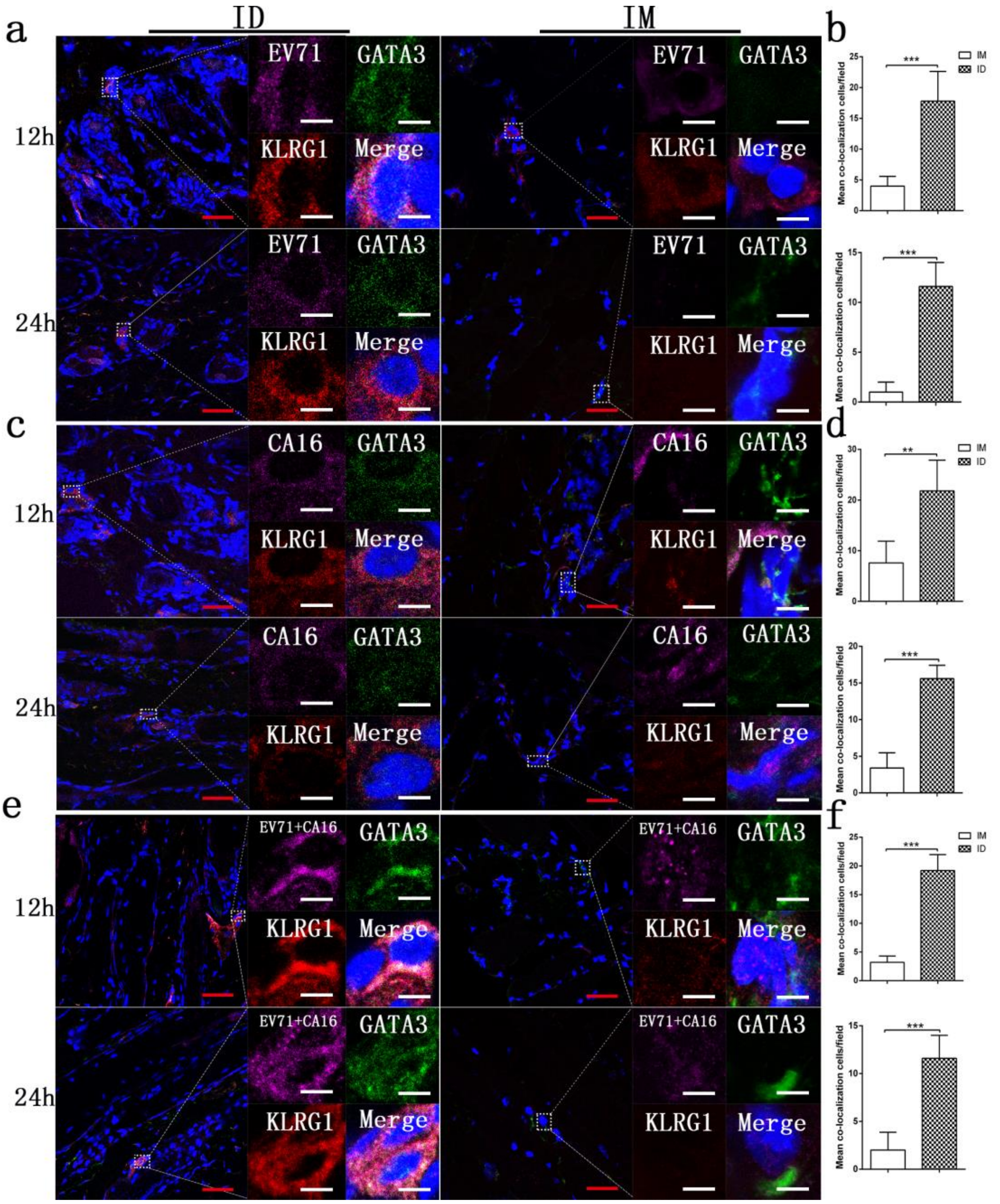

Supplementary Figure 1. Relationships between EV71 or CA16 antigens and ILC2 in local tissues post-inoculation. **a** representative confocal fluorescence images of EV71 expression (purple), GATA3 (green) and KLRG1 (red) via intradermal (ID) or intramuscular (IM) at 12h and 24h post- inoculation of EV71 group. **c** representative confocal fluorescence images of CA16 expression (purple), GATA3 (green) and KLRG1 (red) via intradermal (ID) or intramuscular (IM) at 12h and 24h post- inoculation of CA16 group. **e** representative confocal fluorescence images of EV71+CA16 expression (purple), GATA3 (green) and KLRG1 (red) via intradermal (ID) or intramuscular (IM) at 12h and 24h post- inoculation of EV71+CA16 group. Statistical analysis of counted co-localization cells of **b** EV71, **d** CA16, **f** EV71+CA16 group. The representative confocal fluorescence cells in

white rectangle are shown at 20 × magnification. Red scale bar is 100 μm, white scale bar is 5μm. Data are representatives of three independent experiments (error bars represent SD). Statistical significance were assessed by unpaired t tests (** indicates p < 0.001, *** indicates p < 0.0001)

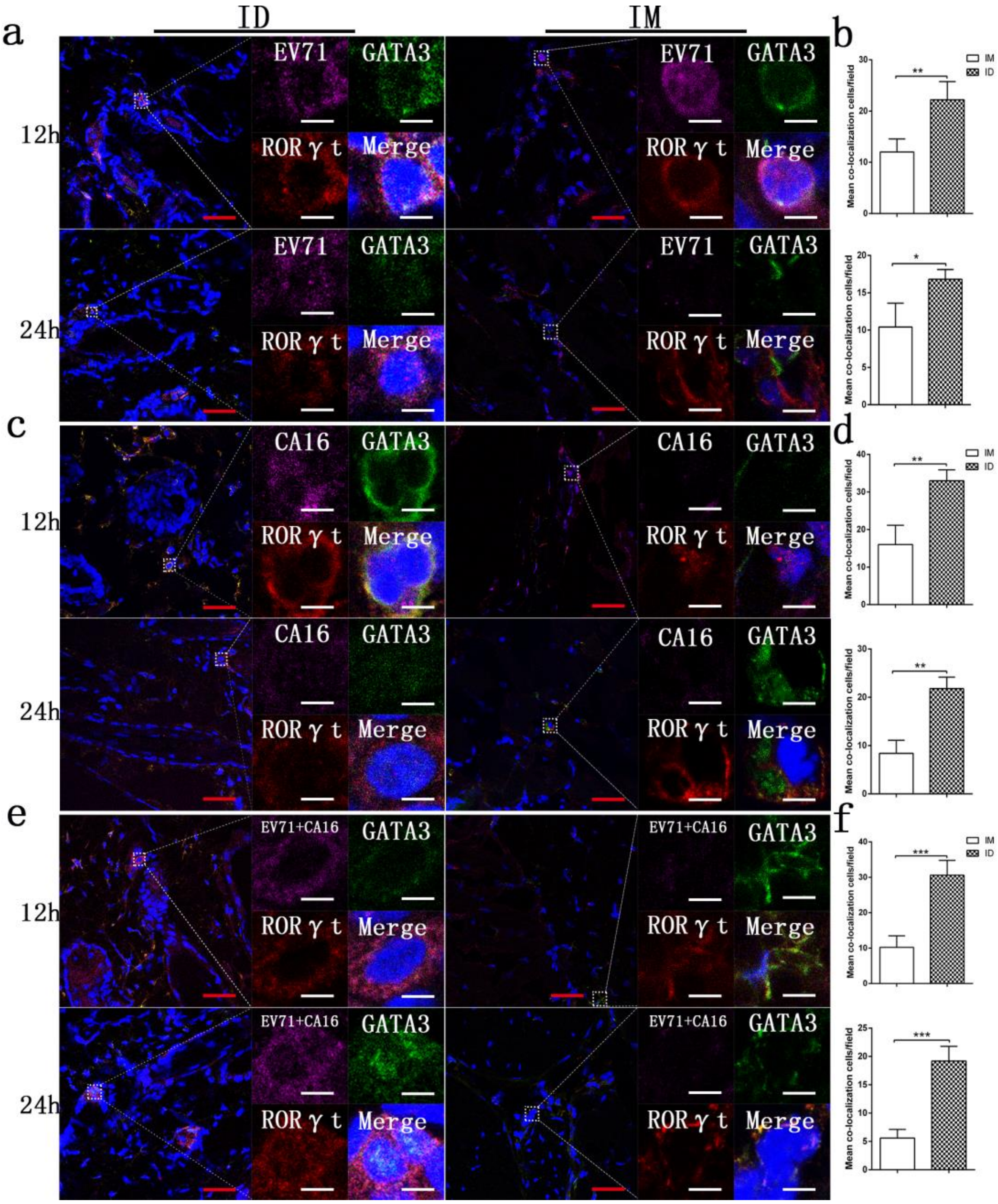

Supplementary Figure 2. Relationships between EV71 or CA16 antigens and ILC3 in local tissues post-inoculation. **a** representative confocal fluorescence images of EV71 expression (purple), GATA3 (green) and ROR γ t (red) via intradermal (ID) or intramuscular (IM) at 12h and 24h post- inoculation of EV71 group. **c** representative confocal fluorescence images of CA16 expression (purple), GATA3 (green) and ROR γ t (red) via

intradermal (ID) or intramuscular (IM) at 12h and 24h post- inoculation of CA16 group. **e** representative confocal fluorescence images of EV71+CA16 expression (purple), GATA3 (green) and ROR γ t (red) via intradermal (ID) or intramuscular (IM) at 12h and 24h post- inoculation of EV71+CA16 group. Statistical analysis of counted co-localization cells of **b** EV71, **d** CA16, **f** EV71+CA16 group. The representative confocal fluorescence cells in white rectangle are shown at 20 × magnification. Red scale bar is 100 μm, white scale bar is 5μm. Data are representatives of three independent experiments (error bars represent SD). Statistical significance were assessed by unpaired t tests (* indicates $p < 0.01$, ** indicates $p < 0.001$, *** indicates $p < 0.0001$)
